# Impact of walking states, self-reported daily walking amount and age on the gait of older adults measured with a smart-phone app: a pilot study

**DOI:** 10.1186/s12877-022-02947-2

**Published:** 2022-03-29

**Authors:** Runting Zhong, Tian Gao

**Affiliations:** School of Business, Jiangnan University, Wuxi, Jiangsu 214122 PR China

**Keywords:** Aging, Gait, Older adults, Smart phone, Walking

## Abstract

**Background:**

Smartphones provide a cost-effective avenue for gait assessment among older adults in the community. The purpose of this study is to explore the impact of walking state, self-reported daily walking amount, and age on gait quality, using a smartphone application.

**Methods:**

One hundred older adult individuals from North China, aged 73.0 ± 7.7 years, voluntarily participated in this study. They performed three walking tests: normal walking, fast walking, and visually impaired walking. Three-dimensional acceleration data for gait were obtained using the smartphone app Pocket Gait. This study used multivariate analysis of variance (MANOVA) to explore the effects of the walking state, self-reported daily walking amount, and age on the step frequency, root mean square (RMS) acceleration, step time variability, regularity, and symmetry.

**Results:**

The walking state, self-reported daily walking amount, and age had statistically significant effects on gait quality. Compared with normal walking, the step frequency, RMS acceleration, variability, and regularity were greater in the fast-walking state, and simulated visually impaired walking did not significantly affect gait quality. Relatively older individuals had a significant decline in gait quality compared to (relatively) younger older adult individuals. Compared with older adults who walked less than 1 km a day, older adults who walked more had better gait quality.

**Conclusions:**

The walking state, self-reported daily walking amount, and age have a significant effect on the gait quality of older adults. Walking with pigmented sunglasses can be used as a training intervention to improve gait performance. Older adult people who walk less than 1 km/day have worse gait quality compared with their counterparts.

## Background

The development of wearable technology provides convenience for measuring gait among older adults in the community [1]. In addition to providing information such as step count, sleep, heart rate, and calorie consumption [2], smartphones can also collect three-dimensional gait data when the user walks naturally, which is convenient for the user to quickly evaluate gait quality in daily life [3–5]. Previous studies have verified the validity and reliability of smartphone-based gait assessment [6, 7] and placing smartphones on the body, bag, or belt is considered most valid for gait assessment [7]. Gait quality monitoring with a smartphone is beneficial for maintaining the health of older adults and is regarded as an incentive for exercise [3].

Gait assessment methods using accelerometer-based technologies are sensitive to detecting age-related changes. Gait quality (gait speed, stride length, frequency, acceleration root mean square, variability, autocorrelation, harmonic ratio) are used as a proxy of health outcomes [1]. Menz et al. [8] found that older adults have lower root mean square (RMS) acceleration, smoothness (harmonic ratio), and higher step time variability compared with younger adults. Kobayashi et al. [9] found that older adults presented lower gait symmetry and regularity than younger adults with trunk accelerometry. Kosse et al. [10] found that, compared to older healthy adults, younger individuals had a more variable, less predictable, and more symmetrical gait pattern according to the trunk gait parameters derived from an iPod. Reference gait parameters measured by a smartphone were established in a laboratory environment for healthy older adults dwelling in communities and older adults were found to have significantly lower step frequency [3]. Acceleration RMS, vertical amplitude variability, and step regularity was found to be lower in pre-frail older adults, compared with non-frail older adults [11]. Stroke patients have significantly lower gait regularity and symmetry compared with healthy adults, according to [12].

When older adults walk, they commonly indulge in dual tasks and these lead to more sensitivity to gait impairment than a single task [13]. Previous studies used different walking states to simulate the daily walking of older adults, such as walking with motor tasks [14], cognitive tasks [11, 14], smartphone usage [15] and wearing smart glasses [16]. It was found that head-worn ‘smart glasses’ had adverse impacts on the gait dynamics of lateral position control [16]. Stepping over hurdles in complex conditions whilst wearing sunglasses led to slower speeds and a higher coefficient of variance compared with the case of not wearing sunglasses [17]. Additionally, visual stimulus traffic lights are an interesting strategy for improving gait performance (gait speed, step length, and cadence) during therapy for older adults [18]. It is important to examine gait quality in different challenging conditions, in order to understand which walking state is beneficial to improving older adults’ gait performance because this has the potential to be used as a rehabilitation therapy in daily life. Therefore, we used different conditions (normal vs. fast vs. visually impaired) to simulate the walking states in older adults’ daily life. It is our primary objective to explore the influence of walking states on the gait quality of older adults.

Walking is an inexpensive and well accepted form of exercise among older adults. Older Asians generally walk more than older white Americans, mainly for transportation and leisure needs [19]. Studies have also used walking as an intervention method, such as Nordic walking [20], walking regularly in an outdoor setting [21], lateral walking [22], treadmill walking [23] and backward walking [24]. Sedentary, older adult people and active older adult individuals of the same age have a significant difference in gait quality, in terms of the acceleration amplitude and angular velocity at the lumbar (RMS) [20]. Other significant differences are found in: the distribution (skewness), quantified from the vertical and Euclidean norm of the lumbar acceleration; the complexity (sample entropy) of the mediolateral lumbar angular velocity and the Euclidean norm of the shank acceleration and angular velocity; the regularity of the lower limbs; the spatiotemporal parameters (walking speed and stride length) and the variability of stance and stride durations [20]. Rocha et al. [21] found that, compared with active older adults, the sedentary older adults prefer a slower pace when walking for a long time on a treadmill. However, there was no significant difference in the variability and changes over time were similar in sedentary and active older adults. Tudor-Locke et al. [25] suggested that an average of 2,000–9,000 steps/day is sufficient for healthy older adults, and an average of 1,200–8,800 steps/day is sufficient for the special population. During the Coronavirus disease-2019 (COVID-19) lockdown, a sharp decline in daily step counts was found (24/01/2020) by an average of 3,796 steps, while the average daily steps were above 8,000 among 815 adults in Shanghai prior to the lockdown [26]. An important question is whether the amount of daily walking has a significant effect on the gait quality of the older adults.

In this study, we used a self-developed smartphone app, Pocket Gait, to measure the gait parameters of older adults in different walking states. As smartphone ownership among older adults is increasing [3], it is beneficial to utilise a smartphone app to track older adults’ gait quality in daily life and promote rehabilitation training. To our knowledge, no prior studies have examined the relationship between older adults’ lifestyle (i.e. self-reported daily walking amount, walking states) and gait quality with a cost-effective smartphone app. Thus, the objective of this study was to explore the impact of the walking state, self-reported daily walking amount, and age on gait quality of older adults with the smart-phone app. The results can help identify groups that are more vulnerable to gait quality decline and need rehabilitation intervention. We hypothesised that older adults have better gait quality in fast-walking speed and worse gait quality in a simulated visually-impaired walking state, compared with normal-walking. In addition, we hypothesised that older adults who walk more have better gait quality compared with sedentary older adults of the same age.

## Methods

### Participants

In March 2021, we invited 100 older adults to participate in the study in communities, parks, and nearby nursing homes in Xingtai, Hebei Province, China. The older adults were recruited through random invitations on the street and with the consent of the nursing home. The inclusion criteria were as follows: (1) age ≥ 60 years, (2) ability to walk independently without assistive tools, and (3) basic communication skills. The exclusion criteria were: (1) unwilling to participate in the experiment, (2) a hunchback, that would influence the pocket placement. This study was approved by the ethics committee of the authors’ university, and informed consent forms were signed by the participants before the experiment. All methods were performed in accordance with the relevant guidelines and regulations.

Our primary hypothesis was that a difference in gait performance would be observed between walking states (normal vs. fast vs. visually impaired). A prior power analysis was performed for the sample size using G*Power [27]. It was estimated that a minimum sample of 43 was required for a statistical power of 0.95 (effect size f = 0.25, $\alpha$ = 0.05, correlation among repeated measures = 0.5). Secondary analysis to compare the gait parameters between age and different self-reported daily walking amounts was exploratory. Hence, we did not conduct sample size calculations for this comparison.

### Design

In the experiment, we used a three walking state (normal walking, fast walking, and visually impaired walking) by two age group (younger older adults and older older adults) by four self-reported daily walking amount (within 1 km, 1–3 km, 3–5 km, 5 km and above) mixed experimental design. The independent variables of this study were: the walking state, age, and the self-reported daily walking amount. The walking state was a within-group variable and the self-reported daily walking amount and age group were between-group variables. The dependent variables were the gait parameters measured using the smartphone app Pocket Gait, including step frequency, RMS acceleration, step time variability, step regularity, and step symmetry (see Table 1).

**Table 1:** Gait parameters collected by the smart phone app in this study

Gait parameters	Calculation
Step frequency (Hz)	The step frequency is calculated by fast Fourier transform. The original vertical acceleration is low-pass filtered, and the frequency corresponding to the peak of the power spectrum is the step frequency [28]. Step frequency is found to be sensitive to age, according to [10]
RMS Acceleration (m/s^2^)	RMS acceleration represents an index of the average amplitude of acceleration in a walking test. It is calculated as RMS = $\sqrt{\frac{{\int }_{{t}_{1}}^{{t}_{n}}{a\left(t\right)}^{2}dt}{{t}_{n}-{t}_{1}}}$ *a(t)* represents the acceleration data at time *t*, ${t}_{1}$ and ${t}_{n}$ represent the start time and end time of data collection, respectively. The RMS acceleration is a proxy of the gait speed [11, 29]
Step time variability	Step time variability = [$\frac{{t}_{SD}}{{t}_{MEAN}}$]$\times 100{\%}$ ${t}_{SD}$ represents the standard deviation of each time step in a walking test and ${t}_{MEAN}$ represents the average time per step. Step time variability is associated with frailty [30], fatigue [31, 32] and falls [33]
Step regularity	Step regularity ${D}_{1}$ is calculated as the autocorrelation coefficient peak near one step. The autocorrelation coefficient peak near one stride (two steps) is recorded as the stride regularity ${D}_{2}$. A higher value of step regularity indicates a greater degree of balance [28]. Step regularity is sensitive to frailty [11] and discriminates between stroke patients and healthy participants [12]
Step symmetry	The symmetry is calculated as the ratio of ${D}_{1}$ to ${D}_{2}$. If ${D}_{2}$> ${D}_{1}$, it is calculated as ${D}_{1}$/${D}_{2}$; if ${D}_{1}$> ${D}_{2}$, it is calculated as ${D}_{2}$/${D}_{1}$. If the symmetry value is closer to 1, it is more symmetrical [34]. Step symmetry is useful to discriminate between stroke patients and healthy participants [12]

### Apparatus

**Fig. 1:**
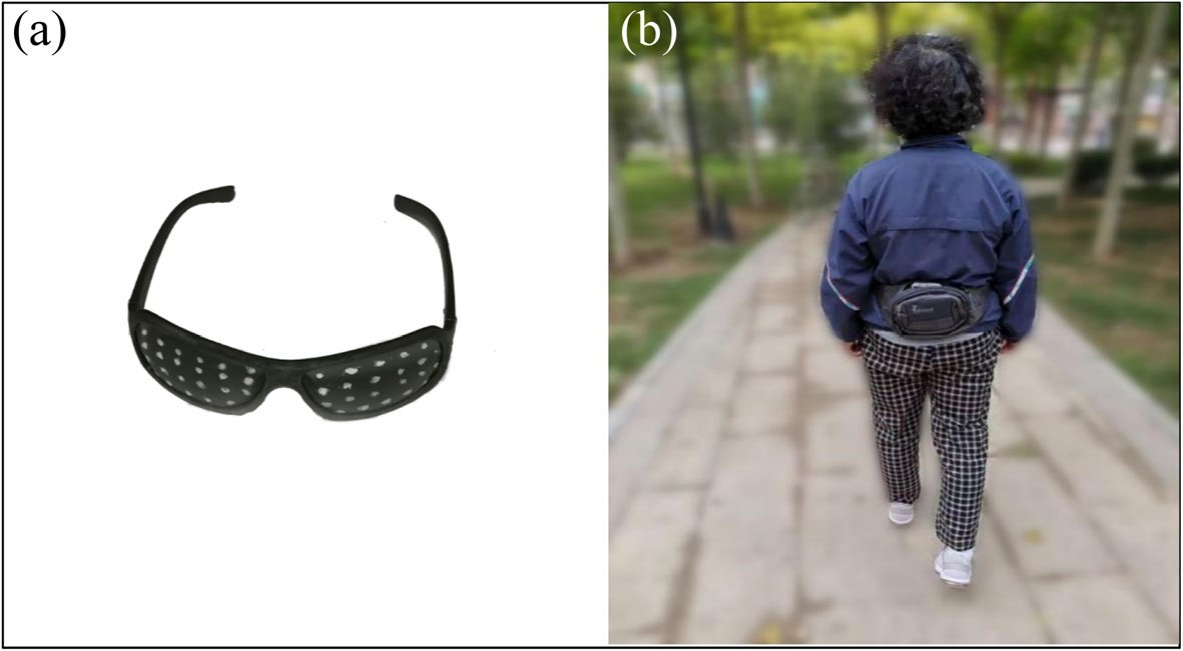
**a** Pigmented sunglasses to simulate visually impaired walking. **b** A participant wore a smartphone in the L3 region of the lower back

The gait assessment system consisted of a smartphone (Huawei Honor v20, Android 9 system, HUAWEI Co. Ltd., China) with the Pocket Gait app. The development and evaluation of the app is described elsewhere [3]. The participants wore the smartphone in the L3 region of the lower back because the waist is sensitive to the stability of the gait. The pocket was placed tightly on the back with an elastic band and the smartphone was fixed with a stick band to avoid shifting. The data quality was checked by checking the graph of walking using the app after a gait trial. Figure 1a presents the pigmented sunglasses and smartphone used at the study sites. The sunglasses were dotted with white paint dots to simulate the highly blurred vision of people looking down the road. The walking trials were carried out outdoors, on level ground in a park, community and nursing home in a relatively quiet environment, as presented in Fig. 1b. The participants took turns in the experiment. When an older adult took a gait trial, others kept their distance, to avoid interference.

### Procedure

We invited the participants to undergo a gait test and the measurement collection tool was an Android smartphone with the Pocket Gait application installed [3].

Then, we asked the participants to complete three independent walks of 40 s, in sequence. The Pocket Gait app was designed to collect 40 s of straight walking when the participant heard the voice instruction of “start walking” given by the app. But the first 5 s gait data were excluded because we would like to eliminate the effects of acceleration at the beginning of walking. The participant would hear “stop walking” at the 40th second. Therefore, the gait data included for data analysis were from the 5th second to the 40th second. In the normal walking state, the participants walked at their most comfortable daily speed. In the fast-walking state, the participants walked at the fastest speed within their ability. Under the visually impaired walking state, the participants walked at their most comfortable speed while wearing pigmented sunglasses to simulate the walking state when their vision was blurred. Each gait task was carried out for one trial.

**Fig. 2:**
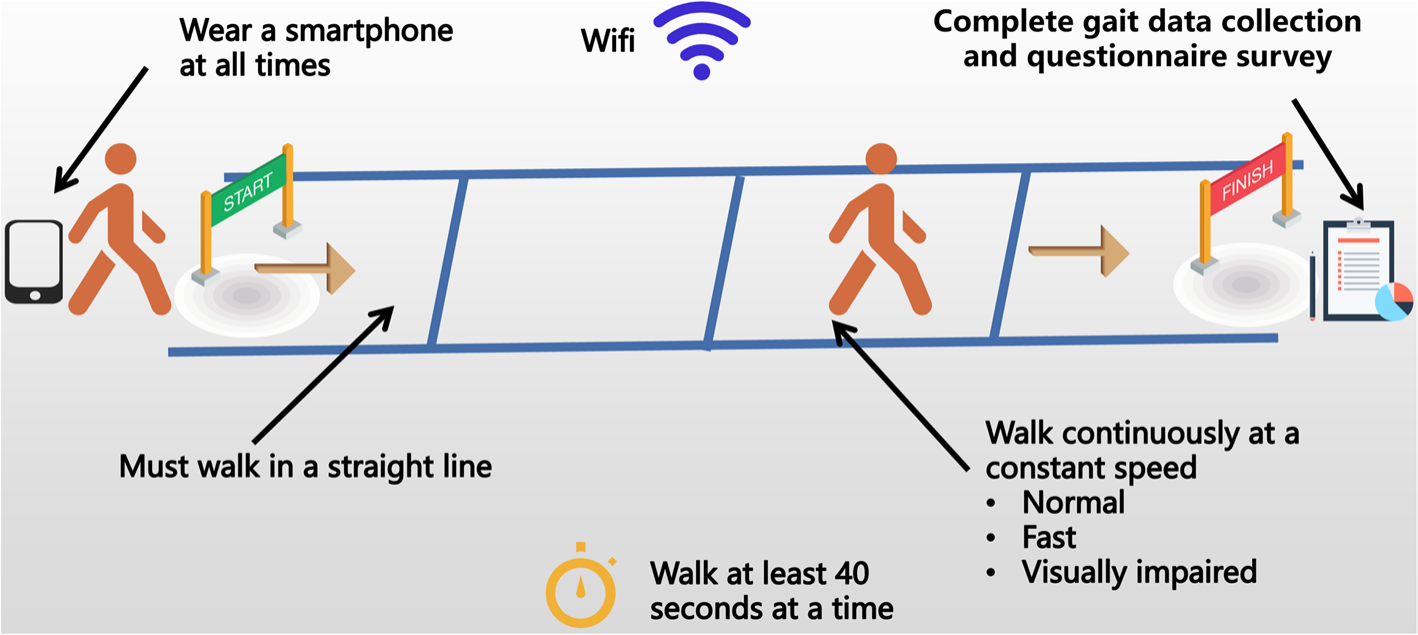
Gait assessment under three walking states: normal, fast, and visually impaired

After the three walks were completed, the researchers used a structured questionnaire to investigate the personal information of the participants, including age, sex, self-reported walking ability, fall history in the past year, fear of falling, medical conditions, and self-reported daily walking amount. We asked the participant, “How do you assess your walking ability?” to measure the walking ability with a 5-point Likert scale (1 = not flexible at all, 3 = neutral, 5 = very flexible). The fall history in the past year was assessed by asking, “How many times did you fall in the last year?” The fear of falling was assessed by asking, “Are you afraid of falling, in your daily life?” with the answer options being: Almost never, Always and Occasionally. Falls in this study were defined as “unintentionally coming to rest on the ground” [29]. We asked the participant, “Do you have the following diseases?” to measure the older adult’s medical conditions. We asked the participant, “How far do you go every day?” to measure the self-reported daily walking amount. Four options were used to classify the participants’ self-reported daily walking amount: 1 km (km) and below, 1–3 km, 3–5 km, and 5 km and above. We used the kilometre as the measurement unit in the questionnaire because, in China, older adults were accustomed to using kilometres as a measurement of distance. Chinese older adults used WeRun (a fitness plugin of Wechat) to record the steps they took each day [2]. WeRun imports step count data from a smartphone’s built-in accelerometer [35]. Therefore, we could estimate their walking distance by checking the steps they walked each day. This question is a classification about whether the older adult is sedentary or physical active. The experiment lasted approximately 20 min. At the end of the experiment, the participant received a gift worth 10 CNY (1.57 USA dollars). A schematic of the experimental process is shown in Fig. 2.

### Data analysis

We downloaded the data from the mobile phone to the computer and then used the MATLAB signal processing toolbox (version 2019b, The MathWorks Inc., Natick, MA, USA) to process the three-dimensional acceleration data. The vertical acceleration was selected for gait analysis because it has strong periodicity. The sampling rate for the app was around 40 Hz. It is the highest mode in the specifications for an Android smartphone, which is SENSOR_DELAY_FASTEST [3]. Acceleration data were interpolated to a constant sampling rate of 100 Hz. The data were de-trended and filtered using a fifth-order low-pass Butterworth filter with a 12.5 Hz cut-off frequency [36]. We compiled the calculation formula for the gait parameters and converted the original acceleration signal into gait parameters (Table 1). The dataset and app in this study can be accessed at [37].

Multivariate analysis of variance was used to explore the influence of the walking status, self-reported daily walking amount, and age group on gait quality, including the step frequency, RMS acceleration, step time variability, step regularity, and symmetry. Mauchly’s sphericity test was used to check the data. If the sphericity was violated, the Greenhouse–Geisser corrected variance analysis was performed. Otherwise, variance analysis was performed under the assumption of sphericity. To interpret the significant main effects, a post-hoc test (Bonferroni method) was performed. Statistical analyses were conducted using SPSS version 26.0 (IBM, Armonk, NY, USA), and the significance level was set at *P* < 0.05. The effect sizes were calculated using partial eta square ${\eta }_{p}^{2}$ (${\eta }_{p}^{2}$ = 0.01 was considered as small, 0.09 as medium and 0.25 as large) [38].

## Results

### Participant characteristics

The participant characteristics are shown in Table 2. The study divided the participants into two groups according to the median age of 72 years: younger older adults (≤ 72 years) and older older adults (> 72 years). There were no significant differences between the two groups, in terms of sex, self-reported walking ability, fall history in the past year, whether they were worried about falling, medical conditions, or the self-reported daily walking amount.

**Table 2:** Participant characteristics for younger and older older adults in this study (*n* = 100)

Variables	All (*n* = 100)	Younger (*n* = 53)	Older (*n* = 47)	*P*
**Sex**				
Male	56(56%)	33(62.3%)	23(48.9%)	0.23
Female	44(44%)	20(37.7%)	24(51.1%)	
**Age (SD)**	73.0 (7.7)	67.1 (3.9)	80.0 (5.1)	< 0.001
**Self-reported walking ability (SD)**^**a**^	3.3 (1.2)	3.4 (1.2)	3.2 (1.2)	0.36
**Fall history in the past year**				
Yes	18(18%)	10(18.9%)	8(17.0%)	0.81
No	82(82%)	43(81.1%)	39(83.0%)	
**Fear of falling**				
Almost never	66(66%)	39(73.6%)	27(57.4%)	0.16
Always	14(14%)	7(13.2%)	7(14.9%)	
Occasionally	20(20%)	7(13.2%)	13(27.6%)	
**Medical conditions**				
Chronic diseases (hypertension, hyperglycaemia, hyperlipemia)	51(51%)	22(41.5%)	29(61.7%)	0.064
Bone and nerve diseases	28(28%)	19(35.8%)	9(19.1%)	
Cardiopulmonary disease	23(23%)	10(18.9%)	13(27.6%)	
Eye diseases	11(11%)	3(5.7%)	8(17.0%)	
Lower extremity muscle diseases	8(8%)	5(9.4%)	3(6.4%)	
None	22(22%)	15(28.3%)	7(14.9%)	
**Self-reported daily walking amount**				
1 km and below	29(29%)	13(24.5%)	16(34.0%)	0.58
1–3 km	26(26%)	13(24.5%)	13(27.7%)	
3–5 km	23(23%)	13(24.5%)	10(21.3%)	
5 km and above	22(22%)	14(26.4%)	8(17.0%)	

The categorical variables sex, self-reported walking ability, fall history, and fear of falling were analysed using the chi-squared test, and continuous variables such as age and self-reported walking ability were analysed using one-way analysis of variance

^a^5-point Likert scale, where 1 = not flexible at all and 5 = very flexible

### Impact of walking state, self-reported daily walking amount and age on gait quality

For the validity of the research, we eliminated the missing data of eight participants caused by data transmission problems during the experiment and only retained the valid data of 92 participants.

### Step frequency

As presented in Table 3, the walking state has a significant effect on the step frequency (*F*_(1.73, 145.05)_ = 126.22, *P* < 0.001, ${\eta }_{p}^{2}=0.60$). The step frequency in each walking state is significantly different (*Ps* < 0.001); the step frequency of normal walking is the smallest, and the step frequency of fast walking is the largest. The age (*F*_(1,84)_ = 1.91, *P* = 0.17, ${\eta }_{p}^{2}=0.02$) and self-reported daily walking amount(*F*_(3,84)_ = 1.37, *P* = 0.26, ${\eta }_{p}^{2}=0.05$) have no significant effect on the step frequency.

**Table 3:** Statistics of step frequency (Hz) (*n* = 92)

**Variables**	***M***	***SD***	**95%*CI***	***F***	***P***	${\eta }_{p}^{2}$
**Walking state**						
Normal	1.84	0.16	1.80–1.87	126.22	< 0.001	0.60
Fast	2.02	0.19	1.98–2.06			
Visually impaired	1.88	0.16	1.85–1.91			
**Age**						
Younger (*n* = 48)	1.92	0.15	1.88–1.97	1.91	0.17	0.02
Older (*n* = 44)	1.90	0.16	1.85–1.94			
**Self-reported daily walking amount**						
1 km and below (*n* = 26)	1.89	0.15	1.83–1.95	1.37	0.26	0.05
1–3 km (*n* = 26)	1.92	0.15	1.86–1.98			
3–5 km (*n* = 19)	1.88	0.15	1.81–1.95			
5 km and above (*n* = 21)	1.97	0.16	1.90–2.03			

### RMS acceleration

As presented in Table 4, there is a significant interaction effect between the walking state and self-reported daily walking amount (*F*_(4.70,131.65)_ = 2.64, *P* = 0.03, ${\eta }_{p}^{2}=0.09$). Under the normal walking state, the RMS acceleration of the older adults who walked less than 1 km is significantly smaller than that of those who walked 3–5 km (*P* = 0.033) or over 5 km and above (*P* < 0.001). Under the fast-walking state, the RMS acceleration of the older adults with less than 1 km of self-reported daily walking is significantly smaller than that of over 5 km (*P* < 0.001). Under the visually impaired walking state, the RMS acceleration of the older adults with less than 1 km of self-reported daily walking is significantly smaller than that with 3–5 km (*P* = 0.047) and over 5 km (*P* < 0.01).

**Table 4:** Statistics of RMS acceleration (m/s^2^) (*n* = 92)

**Variables**	***M***	***SD***	**95%*CI***	***F***	***P***	${\eta }_{p}^{2}$
**Walking state**						
Normal	1.83	0.49	1.74–1.92	142.67	< 0.001	0.63
Fast	2.63	0.87	2.47–2.79			
Visually impaired	1.97	0.61	1.86–2.09			
**Age**						
Younger (*n* = 48)	2.34	0.53	2.18–2.49	12.13	0.001	0.13
Older (*n* = 44)	1.95	0.53	1.79–2.11			
**Self-reported daily walking amount**						
1 km and below (*n* = 26)	1.77	0.53	1.56–1.97	7.32	< 0.001	0.21
1–3 km (*n* = 26)	2.14	0.52	1.94–2.34			
3–5 km (*n* = 19)	2.18	0.52	1.95–2.42			
5 km and above (*n* = 21)	2.49	0.53	2.25–2.72			
**Walking state** $\times$ **Age**						
Normal (Y)	1.96	0.43	1.84–2.09	3.49	0.04	0.04
Fast (Y)	2.90	0.75	2.68–3.11			
Visually impaired (Y)	2.15	0.55	1.99–2.31			
Normal (O)	1.70	0.44	1.57–1.83			
Fast (O)	2.37	0.77	2.14–2.60			
Visually impaired (O)	1.79	0.56	1.62–1.96			
**Walking state** $\times$ **Self-reported daily walking amount**						
Normal (1 km)	1.52	0.43	1.35–1.69	2.64	0.03	0.09
Fast (1 km)	2.16	0.75	1.86–2.45			
Visually impaired (1 km)	1.63	0.55	1.41–1.84			
Normal (1–3 km)	1.82	0.43	1.65–1.99			
Fast (1–3 km)	2.62	0.74	2.33–2.91			
Visually impaired (1–3 km)	1.98	0.55	1.77–2.19			
Normal (3–5 km)	1.89	0.43	1.69–2.09			
Fast (3–5 km)	2.58	0.75	2.24–2.92			
Visually impaired (3–5 km)	2.08	0.54	1.83–2.33			
Normal (5 km and above)	2.09	0.44	1.90–2.28			
Fast (5 km and above)	3.17	0.77	2.84–3.50			
Visually impaired (5 km and above)	2.19	0.56	1.95–2.44			

There is a significant interaction effect between the walking state and age (*F*_(1.57,131.65)_ = 3.49, *P* = 0.04, ${\eta }_{p}^{2}=0.04$). Under the three walking states, the RMS acceleration of the younger older adults is significantly larger than that of the older older adults (*Ps* < 0.05).

### Step time variability

As presented in Table 5, there is a significant interaction effect between the walking status and age (*F*_(1.83,153.90)_ = 4.44, *P* = 0.02, ${\eta }_{p}^{2}=0.05$). In the normal walking state, the variability of the younger older adults is significantly higher than that of the older older adults (*P* < 0.01). In the fast-walking state, there is no significant difference in variability between the younger and older older adults (*P* = 0.71). In the visually impaired walking state, the variability of the younger older adults is significantly higher than that of the older older adults (*P* = 0.03).

**Table 5:** Statistics of step time variability (*n* = 92)

**Variables**	***M***	***SD***	**95%*CI***	***F***	***P***	${\eta }_{p}^{2}$
**Walking state**						
Normal	0.15	0.07	0.13–0.16	9.36	0.001	0.10
Fast	0.18	0.09	0.16–0.20			
Visually impaired	0.14	0.07	0.13–0.16			
**Age**						
Younger (*n* = 48)	0.17	0.05	0.15–0.18	3.61	0.06	0.05
Older (*n* = 44)	0.15	0.06	0.13–0.16			
**Self-reported daily walking amount**						
1 km and below (*n* = 26)	0.17	0.06	0.15–0.19	2.72	0.05	0.09
1–3 km (*n* = 26)	0.14	0.06	0.12–0.16			
3–5 km (*n* = 19)	0.18	0.06	0.15–0.20			
5 km and above (*n* = 21)	0.15	0.05	0.12–0.17			
**Walking state** $\times$ **Age**						
Normal (Y)	0.17	0.07	0.15–0.19	4.44	0.02	0.05
Fast (Y)	0.18	0.09	0.15–0.20			
Visually impaired (Y)	0.16	0.07	0.14–0.18			
Normal (O)	0.13	0.07	0.11–0.15			
Fast (O)	0.18	0.09	0.16–0.21			
Visually impaired (O)	0.13	0.07	0.11–0.15			

Y = younger older adult, O = older older adult

The self-reported daily walking amount has a significant effect on step time variability (*F*_(3,84)_ = 2.72, *p* = 0.05, ${\eta }_{p}^{2}$ = 0.09). However, the post-hoc analysis shows that there is no significant difference in step time variability between the self-reported daily walking amount groups (*Ps* > 0.05).

### Step regularity

As presented in Table 6, the walking status (*F*_(2,168)_ = 18.50, *P* < 0.001, ${\eta }_{p}^{2}$** =** 0.18), age (*F*_(1,84)_ = 4.88, *P* = 0.03, ${\eta }_{p}^{2}$** =** 0.06), and self-reported daily walking amount (*F*_(3,84*)*_ = 8.39, *p* < 0.001, ${\eta }_{p}^{2}$** =** 0.23) have significant effects on the step regularity. There are significant differences in the step regularity in each walking state. The step regularity of normal walking is the smallest, and the step regularity of fast walking is the largest. The step regularity of the younger older adults is significantly greater than that of the older older adults (*P* = 0.03). The step regularity of the older adults with less than 1 km of self-reported daily walking is significantly smaller than those with 1–3 km (*P* = 0.01), 3–5 km (*P* < 0.01), and above 5 km (*P* < 0.001).

**Table 6:** Statistics of step regularity (*n* = 92)

**Variables**	***M***	***SD***	**95%*CI***	***F***	***P***	${\eta }_{p}^{2}$
**Walking state**						
Normal	0.68	0.18	0.65–0.72	18.50	< 0.001	0.18
Fast	0.74	0.18	0.70–0.77			
Visually impaired	0.71	0.17	0.68–0.74			
**Age**						
Younger (*n* = 48)	0.74	0.15	0.70–0.79	4.88	0.03	0.06
Older (*n* = 44)	0.67	0.15	0.63–0.72			
**Self-reported daily walking amount**						
1 km and below (*n* = 26)	0.59	0.15	0.53–0.64	8.39	< 0.001	0.23
1–3 km (*n* = 26)	0.72	0.15	0.66–0.78			
3–5 km (*n* = 19)	0.74	0.15	0.67–0.80			
5 km and above (*n* = 21)	0.79	0.15	0.73–0.86			

### Step symmetry

As presented in Table 7, there is no significant difference in the symmetry of each walking state. The age (*F*_(1,84)_ = 3.99, *P* = 0.05, ${\eta }_{p}^{2}$** =** 0.05) and self-reported daily walking amount (*F*_(3,84)_ = 5.44, *p* < 0.01, ${\eta }_{p}^{2}$** =** 0.16) have a significant effect on symmetry. The symmetry of the younger and older older adults is significantly higher than that of the older older adults (*P* = 0.05). The symmetry of the older adults with less than 1 km of self-reported daily walking is significantly lower than that of the older adults with 1–3 km (*P* < 0.01), 3–5 km (*P* < 0.01), and above 5 km (*P* < 0.01).

**Table 7:** Statistics of step symmetry (*n* = 92)

**Variables**	***M***	***SD***	**95%*CI***	***F***	***P***	${\eta }_{p}^{2}$
**Walking state**						
Normal	0.89	0.13	0.87–0.92	0.28	0.73	< 0.01
Fast	0.90	0.14	0.87–0.93			
Visually impaired	0.89	0.12	0.87–0.92			
**Age**						
Younger (*n* = 48)	0.92	0.10	0.89–0.95	3.99	0.05	0.05
Older (*n* = 44)	0.87	0.11	0.84–0.90			
**Self-reported daily walking amount**						
1 km and below (*n* = 26)	0.82	0.10	0.78–0.86	5.44	< 0.01	0.16
1–3 km (*n* = 26)	0.91	0.10	0.87–0.96			
3–5 km (*n* = 19)	0.92	0.10	0.88–0.97			
5 km and above (*n* = 21)	0.92	0.11	0.87–0.97			

## Discussion

### Principle findings

This study aimed to explore the effect of walking state, self-reported daily walking amount, and age on the gait quality of Chinese older adults with a smartphone app. Gait quality was measured by the step frequency, RMS acceleration, step time variability, regularity, and symmetry. Larger step frequency, RMS acceleration, step regularity, symmetry, and smaller step time variability were considered to be indicators of better gait [1, 3]. In this study, we have inferred that the walking state, self-reported daily walking amount, and age have statistically significant effects on gait quality. In terms of the walking state, simulated visually impaired walking does not significantly affect gait quality. Compared with the older adults who walk less than 1 km a day, the older adults who walk more have better gait quality. Older older adults have a significant decline in gait quality compared to younger older adults, except for step time variability. Compared with a previous laboratory study [3], gait variability in daily life situations is greater, and symmetry and stability are worse, which are consistent with the findings of [39].

The walking state has a significant impact on gait quality. In the fast-walking state, the step frequency is higher, and the RMS acceleration, step time variability, and step regularity are larger, which indicate that fast walking affects the stability of walking. Unlike previous studies, which found that smart glasses had an adverse effect on lateral gait [16, 17], we infer that simulated visually impaired walking does not significantly affect gait quality compared to normal walking, which may be due to human visual adaptability. Therefore, walking with pigmented sunglasses can be used as a cost-effective training intervention to improve gait performance among the older adults.

The self-reported daily-walking amount has a significant effect on the gait quality. Compared with the older adults of the same age who walk less (less than 1 km per day), those who walk more have better gait quality, which is reflected in the greater RMS acceleration, step regularity, and symmetry. A possible explanation is that the older adults who walk more are more physically active and have better posture control. Earlier, it was found that frail and non-frail older adults differed significantly in the number of steps (frail: 1029 steps vs. non-frail: 8409 steps) and total walking time per day (frail: 14 min vs. non-frail: 99.5 min) [40]. Regular physical activity, such as Nordic walking training, may counteract the deterioration of age-related gait quality [20]. This study confirms that walking less than 1 km can potentially affect gait quality, and strengthening daily walking is a good strategy to improve gait quality.

Age has a significant effect on gait. Compared with the younger older adults, the older older adults have smaller RMS acceleration, step regularity, and symmetry. Surprisingly, in this study, we found that the variability in older older adults is smaller than that in younger older adult individuals under the normal walking or visually impaired states. Studies have shown that high variability is a risk factor for falling [33] and frailty [30], and some studies have shown that high or low variability is related to walking speed [16]. However, Kosse et al. [9] found that healthy younger people have greater variability, are more difficult to predict, and have a higher symmetry than older adults. The results of this study are consistent with these findings [10]. Gait variability is not linearly related to age but follows a U-shape. A possible explanation is that older adults walk more conservatively. Therefore, a single variability is not recommended as the only indicator of risk of fall, and multidimensional gait indicators should be considered comprehensively.

### Clinical implications

Gait quality is related to older adults’ life quality and gait assessment is useful for stroke patients, Parkinson’s patients, hemiplegia, cerebral palsy, rheumatoid arthritis, recovery after joint operations and generally healthy adults in the community. The study provides evidence of using a novel geotechnology (i.e. the “Pocket Gait” app) to quantify gait and also discusses the association between older adults’ lifestyle and gait quality measured by the smartphone app. This study provides evidence that older adults who walked less than 1 km had worse quality gait compared with their counterparts with more than 1 km walking distance per day. Thus, this group (less than 1 km of self-reported daily walking per day) should pay attention to gait rehabilitation, such as carrying out Tai Chi. This result is useful for health promotion implications during the COVID-19 pandemic. Government and health professionals should encourage walking among older adults, specifically, more than 1 km per day.

Regarding the positioning of this technology, the “Pocket Gait” app in this study could be used as a tool to track gait quality at home or in the community. It does not require a large digital field, is simple to operate, and is inexpensive. Older adults can learn about their gait quality anytime and anywhere. For healthy adults, the app could serve as a tool to detect status, such as fatigue or frailty. For younger older adults, the app could be used as an incentive for exercise. For older older adults, the app could be used to detect early signs of aging, such as a decline in RMS acceleration, step regularity, and symmetry. For patients with stroke or Parkinson’s disease, the app could serve as an assistive tool for rehabilitation at home and reduce outpatient visits.

### Limitations and future research

A major limitation of this study was the use of self-report for measurement of daily walking distance, which may differ from the actual situation, although there was evidence that used self-reported questionnaires for gait assessment, such as telephone-based mobility assessment questionnaire (TMAQ) [41]. To reduce the bias, we checked the participants’ daily walking steps using the WeRun plugin to estimate their walking distance [2]. According to a previous study, community-dwelling older Chinese have an average stride length of 1.29 m (95% *CI*: 1.22–1.35 m) at normal speed [42]. Therefore, we could estimate their daily walking distance by checking the steps they walked each day (walking distance = stride length $\times$ steps/2). Another potential limitation of this study was that we used a cross-sectional study with a convenience sample. Future studies could collect long-term data to track the rehabilitation progress of the older adults in different time periods (e.g. morning, afternoon vs. evening or before vs. after taking medicine). Second, mobile phones can also be placed in other locations, such as handbags or pockets, to establish gait reference values in other locations. Third, the app could be used as an intervention for public health. Older adults may view their gait report compared with their peers. Future studies could also combine the quantity (e.g. walking steps recorded by WeRun) and gait quality of walking through wearable technologies, as health interventions for the older adults.

## Conclusion

To our knowledge, this is the first study to examine the relationship between older adults’ lifestyle (i.e. self-reported daily walking amount, walking states) and gait quality with a cost-effective smartphone app. We concluded that the walking status has a significant impact on gait quality. Compared with normal walking, the step frequency, RMS acceleration, variability, and regularity are greater in the fast-walking state, and simulated visually impaired walking does not significantly affect gait quality. The amount of self-reported daily walking has a positive effect on the gait quality. Compared with the older adults, who walk less in the same age group (less than 1 km per day), the older adults who walk more have better gait quality, which is reflected in greater RMS acceleration, step regularity, and symmetry. Age has a significant effect on the gait quality. Compared with the younger older adults, the RMS acceleration, step regularity, symmetry, and variability of the older older adults are smaller.

## Data Availability

The datasets analyzed during the current study are available at Zhong, R., & Gao, T. (2022). *Gait Assessment of Chinese Older adults in Daily Environment Using the “Pocket Gait” App*. Mendeley Data, V3. http://dx.doi.org/10.17632/nk4n3jcsk6.3.

## Abbreviations

RMS: Root mean square
MANOVA: Multivariate analysis of variance
M: Mean
SD: Standard deviation
CI: Confidence interval
